# A Rare Encounter of Cervical Tuberculous Lymphadenitis (Scrofula) in an Immunocompetent Adolescent Female: A Case Report

**DOI:** 10.7759/cureus.83428

**Published:** 2025-05-03

**Authors:** Ifeanyi K Uche, Sagar Kansara, Catherine S O'Neal

**Affiliations:** 1 School of Medicine, Louisiana State University Health Sciences Center, New Orleans, USA; 2 Otolaryngology-Head and Neck Surgery, Louisiana State University Health Sciences Center, New Orleans, USA; 3 Clinical Medicine and Infectious Diseases, Our Lady of the Lake Regional Medical Center, Baton Rouge, USA

**Keywords:** extrapulmonary tb, immunosupression, lymphadenopathy, scrofula, tuberculosis

## Abstract

Tuberculosis (TB) remains a major global health concern. While it primarily affects the lungs, TB can also present in extrapulmonary forms, with cervical tuberculous lymphadenitis (CTL) or scrofula being the most common. Herein, we report a case of a 19-year-old female with a six-month history of progressively enlarging left-sided neck swelling. The patient was diagnosed with CTL. Diagnosing extrapulmonary TB can be challenging. In cases like ours, excisional biopsy or fine-needle aspiration (FNA) cytology with acid-fast bacilli (AFB) smear, culture, or polymerase chain reaction (PCR) analysis is critical for diagnosis. Our case highlights the importance of maintaining a high index of suspicion to differentiate mycobacterial infection from other causes of lymphadenopathy, particularly in immunocompetent adolescent patients.

## Introduction

Although rare in the United States, tuberculosis (TB) continues to cause substantial global morbidity and mortality. The incidence of reported TB has decreased gradually in the United States, from 9.6 cases per 100,000 population in 1993 to 2.18 cases per 100,000 in 2020 [1,2]. Pulmonary TB has shown a much steeper decline than extrapulmonary TB; in fact, the percentage of TB cases that are extrapulmonary has been gradually increasing from 15.7% in 1993 to 21.1% in 2021 [2]. Cervical tuberculous lymphadenitis (CTL) or scrofula is the most common extrapulmonary manifestation of TB [3]. CTL is more commonly seen in children, women, minorities, and immunocompromised patients, especially those with human immunodeficiency virus (HIV) [4]. Patients with CTL usually present with neck swelling and have systemic symptoms like fever, night sweats, weight loss, and fatigue, which are often subtle or maybe absent [5]. Of note, CTL is a great imitator and can closely mimic malignancy or other pathological conditions, such as sarcoidosis, lymphoma, or metastatic lymphadenopathy from papillary thyroid carcinoma [6,7]. This resemblance often leads to misdiagnosis and unnecessary delays in diagnosis and the initiation of treatment.

## Case presentation

A 19-year-old woman with no significant past medical history presented with a progressively enlarging, painful swelling on the left side of her neck that had been present for six months. She was not taking any medications at the time and had never received the Bacillus Calmette-Guérin (BCG) vaccine. She reported having had an upper respiratory tract infection several months earlier, which preceded the development of right-sided cervical lymphadenopathy that subsequently resolved. However, the left-sided swelling persisted. She complained of night sweats but denied other systemic symptoms such as fever, chills, cough, or weight loss. The upper respiratory infection occurred after she had spent a week working in New York at a fashion-related event. At the time, she denied any sick contacts or exposure to pets or animals but did report recent travel outside the United States.

**Figure 1:**
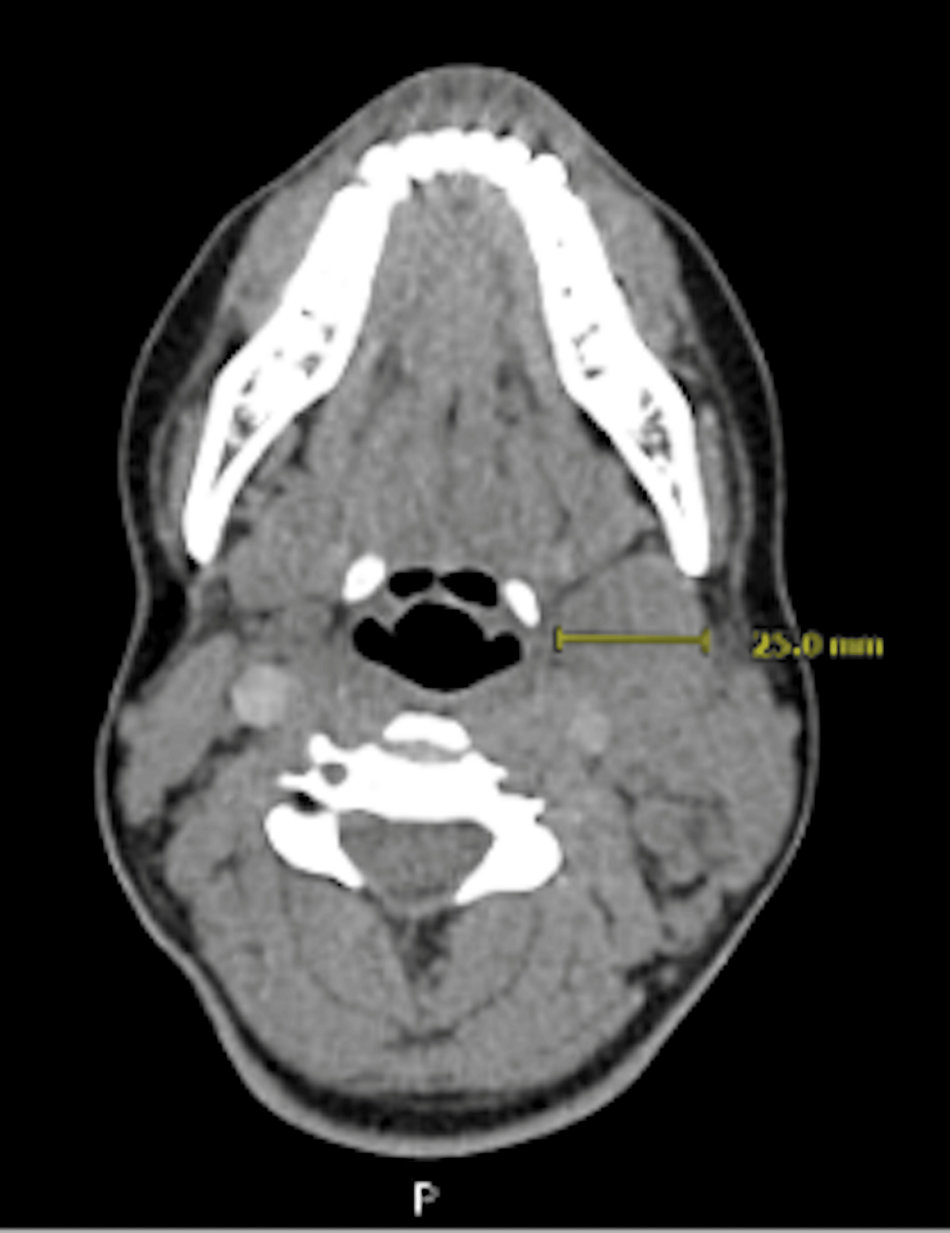
Representative CT image showing swelling on the left side of the patient’s neck Axial CT image of the neck with contrast showing large level II matted left cervical adenopathy, measuring 2.5 cm in greatest dimension. CT, computed tomography

**Figure 2:**
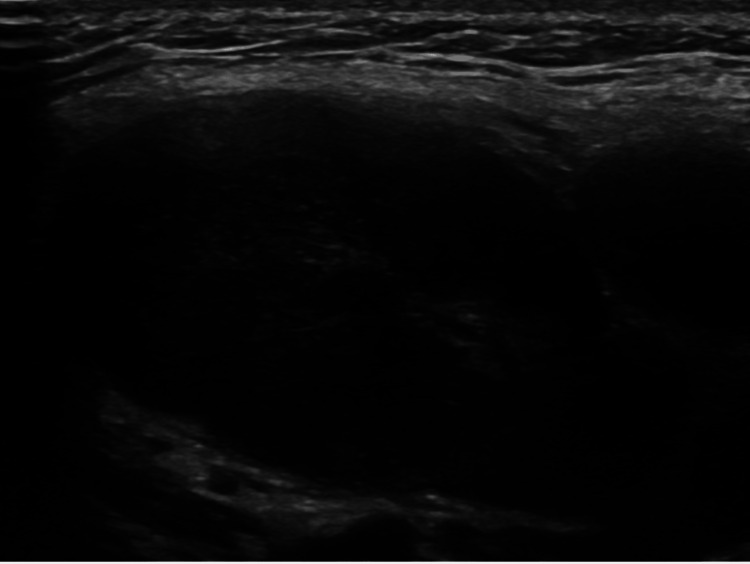
Representative ultrasound image showing swelling on the left side of the patient’s neck Ultrasound image of the left neck revealing a large cystic mass at level II, measuring 2.5 x 2 cm.

The patient was initially referred to Otolaryngology-Head and Neck Surgery for evaluation. Physical examination revealed a firm, approximately 2 cm, level II left cervical lymph node. Flexible endoscopy showed no abnormalities. A contrast-enhanced computed tomography (CT) scan of the neck demonstrated multiple enlarged left cervical lymph nodes, several of which appeared suppurative or necrotic (Figure 1), raising suspicion of a lymphoproliferative disorder, such as lymphoma. As a result, an ultrasound-guided fine-needle aspiration (FNA) (Figure 2) and an excisional lymph node biopsy were performed.

FNA results were negative for Gram stain, anaerobes, and fungal organisms. The acid-fast culture of the node was initially negative. The cytologic evaluation showed no evidence of malignancy but revealed both necrotizing and non-necrotizing granulomatous inflammation. Based on these findings, the patient was referred to Infectious Disease and Rheumatology for further evaluation.

A chest X-ray was normal. Infectious disease workup, including tests for HIV, hepatitis C, Toxoplasma gondii, Treponema pallidum (syphilis), Epstein-Barr virus, Bartonella, and fungal infections such as Blastomyces and Histoplasma, was negative. However, her QuantiFERON-TB test was positive. PCR analysis of the FNA sample was also positive for Mycobacterium tuberculosis, and after four weeks, acid-fast bacilli (AFB) culture confirmed the growth of the Mycobacterium tuberculosis complex, establishing the diagnosis of CTL, also known as scrofula.

The patient was started on a six-month treatment regimen consisting of pyrazinamide 1000 mg, ethambutol 800 mg, rifampin 600 mg, and isoniazid 300 mg. Follow-up was arranged through the local public health TB clinic. However, the patient was subsequently lost to follow-up.

## Discussion

Mycobacterium tuberculosis remains a significant global health issue and is one of the deadliest infectious diseases worldwide. While TB is more prevalent in low- and middle-income countries, it has been increasing in developed nations due to factors such as the rise of immunosuppressive conditions, particularly HIV/AIDS, and increased immigration from high-incidence regions [8].

TB primarily affects the lungs, but extrapulmonary TB can occur, with CTL, or scrofula, being the most common form [3,8]. In medieval Europe, scrofula was referred to as the "King's Evil" because newly crowned kings were believed to have the ability to cure the disease through their touch, a belief that persisted until the 18th century [9]. Scrofula typically presents as a painless, enlarging neck mass, averaging 3 cm in size, over a period of three to four weeks, although it has been reported to persist for up to eight years [10]. Extrapulmonary TB is more common in females, young children (<15 years of age), older adults (>65 years of age), and patients with HIV [11,12].

Our patient resides in Louisiana, where 10.1% of TB cases in 2020 involved both pulmonary and extrapulmonary sites, according to the Louisiana Annual Tuberculosis Report 2021. In 2023, a total of 100 cases were reported in Louisiana, with a rate of 2.19 cases per 100,000 people. In countries with very low TB incidence, diagnosis can be challenging and may delay the initiation of appropriate treatment [13]. Contributing factors include limited public awareness and knowledge of TB symptoms, leading to delayed health-seeking behavior; socioeconomic barriers such as low income, unemployment, rural residence, and limited access to healthcare facilities; delays in specialist referrals and diagnostic procedures; and a low index of clinical suspicion among healthcare providers.

CTL, or scrofula, is typically accompanied by fever, chills, malaise, and weight loss. However, in our case, the patient did not exhibit any of these systemic symptoms. Similar cases have been reported previously [14,15]. In one case, the patient had a history of TB in childhood, while in another, the patient was a 46-year-old woman from El Salvador. Nevertheless, in cases like ours, where a fluctuant neck mass is present in an immunocompetent patient with no obvious systemic symptoms, extrapulmonary TB should remain a high clinical suspicion, even if initial AFB cultures are negative. It is important to note that AFB cultures used to confirm TB or other mycobacterial infections can take up to eight weeks to show positive results [16]. Therefore, follow-up evaluations are essential, even if initial results are negative or after four weeks of incubation. Obtaining cultures during an FNA is critical in assisting with the diagnosis of lymphadenopathy.

## Conclusions

Our case highlights the importance of considering extrapulmonary TB, particularly CTL or scrofula, in the differential diagnosis of neck masses, even in immunocompetent patients. The patient in our case presented with a progressively enlarging left-sided neck mass and minimal systemic symptoms. Prompt diagnosis and initiation of the standard TB treatment regimen led to a favorable outcome.
